# RECQL5 Controls Transcript Elongation and Suppresses Genome Instability Associated with Transcription Stress

**DOI:** 10.1016/j.cell.2014.03.048

**Published:** 2014-05-22

**Authors:** Marco Saponaro, Theodoros Kantidakis, Richard Mitter, Gavin P. Kelly, Mark Heron, Hannah Williams, Johannes Söding, Aengus Stewart, Jesper Q. Svejstrup

**Affiliations:** 1 Mechanisms of Transcription Laboratory, Clare Hall Laboratories, Cancer Research UK London Research Institute, South Mimms, EN6 3LD, UK; 2 Bioinformatics and Biostatistics Group, Cancer Research UK London Research Institute, 44 Lincoln’s Inn Fields, London WC2A 3LY, UK; 3 Gene Center and Center for Integrated Protein Science Munich (CIPSM), Ludwig-Maximilians-Universität München, Feodor-Lynen-Strasse 25, 81377 Munich, Germany

## Abstract

RECQL5 is the sole member of the RECQ family of helicases associated with RNA polymerase II (RNAPII). We now show that RECQL5 is a general elongation factor that is important for preserving genome stability during transcription. Depletion or overexpression of RECQL5 results in corresponding shifts in the genome-wide RNAPII density profile. Elongation is particularly affected, with RECQL5 depletion causing a striking increase in the average rate, concurrent with increased stalling, pausing, arrest, and/or backtracking (transcription stress). RECQL5 therefore controls the movement of RNAPII across genes. Loss of RECQL5 also results in the loss or gain of genomic regions, with the breakpoints of lost regions located in genes and common fragile sites. The chromosomal breakpoints overlap with areas of elevated transcription stress, suggesting that RECQL5 suppresses such stress and its detrimental effects, and thereby prevents genome instability in the transcribed region of genes.

## Highlights

• RECQL5 is a general RNAPII elongation factor
• RECQL5 reduces the elongation rate while decreasing pausing and arrest events
• Loss of RECQL5 results in genome instability in genes and at common fragile sites
• Incidents of genome instability colocalize with pause and arrest events

## Graphical Abstract

**Figure:**
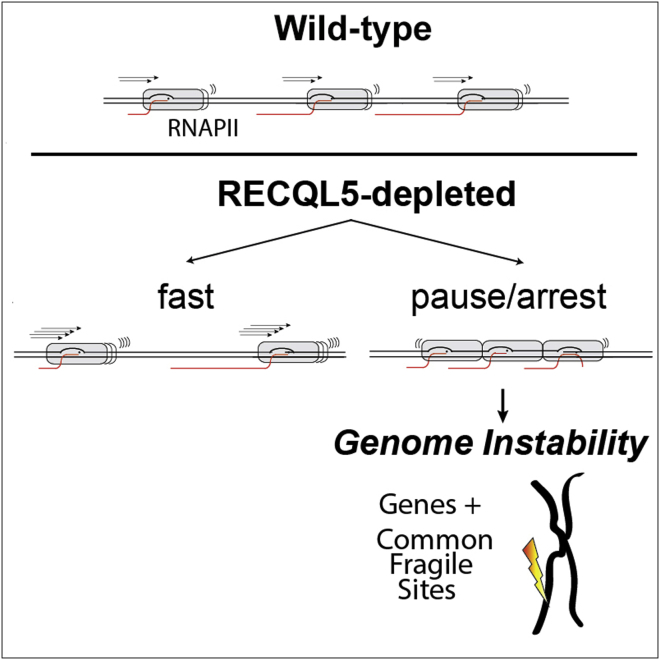

## Teaser

Rapid elongation by RNA polymerase II leads to increased transcriptional stress including a high incidence of pausing and arrests, which correlates with sites of genomic instability. RECQL5 modulates the rate of transcription, mitigating both the stress and instability effects.

## Introduction

Over the last decade it has become increasingly evident that transcription is closely integrated with other DNA-related processes, such as chromatin dynamics, DNA replication, and repair. Indeed, the essential process of expressing genes comes at a cost: the movement of RNA polymerases through DNA is associated with genome instability (Aguilera and Gómez-González, 2008; Helmrich et al., 2013), and RNAPII stalling, pausing, arrest, and/or backtracking (hereafter collectively referred to as transcription stress) generates a cellular response akin to the DNA damage response (Wilson et al., 2013).

Transcribing polymerases are potent modulators of other DNA-related processes, such as DNA replication. For example, transcription-associated DNA recombination involves clashes between transcription and replication, and transcription is also associated with mutagenesis and contraction of CAG repeats, as well as breaks at chromosome fragile sites (Aguilera and Gómez-González, 2008; Helmrich et al., 2013). However, the mechanisms underlying transcription-associated genome instability remain largely obscure, and little is known about factors that might have evolved to counteract it.

The RECQ proteins constitute a family of conserved DNA helicases that are important for maintaining genome stability, from bacteria to humans (Chu and Hickson, 2009). The human genome encodes five RECQ family members: RECQL (RECQ1), BLM (RECQ2), WRN (RECQ3), RECQL4, and RECQL5. Mutations in three of these, namely *BLM*, *WRN*, and *RECQL4*, give rise to hereditary disorders associated with cancer predisposition and premature aging (Bloom’s, Werner’s, and Rothmund-Thomson’s syndrome, respectively) (Chu and Hickson, 2009). Moreover, *Recql5* knockout mice exhibit elevated levels of sister chromatid exchange and are predisposed to various types of cancer (Hu et al., 2005; Hu et al., 2007), suggesting that RECQL5 is important for maintaining genome stability as well. Indeed, RECQL5 has been implicated in the prevention of replication fork collapse and the accumulation of DNA double-strand breaks (Hu et al., 2009; Popuri et al., 2012) and is also somehow involved in the suppression or removal of endogenous DNA damage as well as psoralen-induced interstrand crosslinks (Li et al., 2011; Tadokoro et al., 2012; Ramamoorthy et al., 2013). Concomitant mutation in *RECQL5* and another *RECQ* family member typically has a more severe effect than mutation in just one (see, for example, Hu et al., 2005; Popuri et al., 2013), suggesting that RECQL5 is active in pathways distinct from those governed by other RECQ members. However, despite RECQL5 being phenotypically implicated in the preservation of genome integrity at several levels, the mechanistic basis for its function remains poorly understood.

RECQL5 is unique among the RECQ family by interacting with RNAPII, and it harbors two RNAPII interaction domains, which are relevant for the phenotypes of different RECQL5-deficient metazoan cell types (Aygün et al., 2008; Izumikawa et al., 2008; Islam et al., 2010; Li et al., 2011). Intriguingly, transcription reactions reconstituted with pure general transcription factors and RNAPII suggest that RECQL5 might act as an inhibitor of transcription (Aygün et al., 2009). However, the physiological relevance, if any, of this surprising observation has not been investigated. Likewise, the mechanistic relationship between the effects of *RECQL5* mutation on genome integrity and RNAPII transcription has also remained unclear. Here, we report that RECQL5 has genome-wide effects on transcript elongation and that it suppresses genome rearrangements associated with common fragile sites and transcription stress in the body of genes.

## Results

### RECQL5 Affects RNAPII Progression Genome-wide

**Figure 1:**
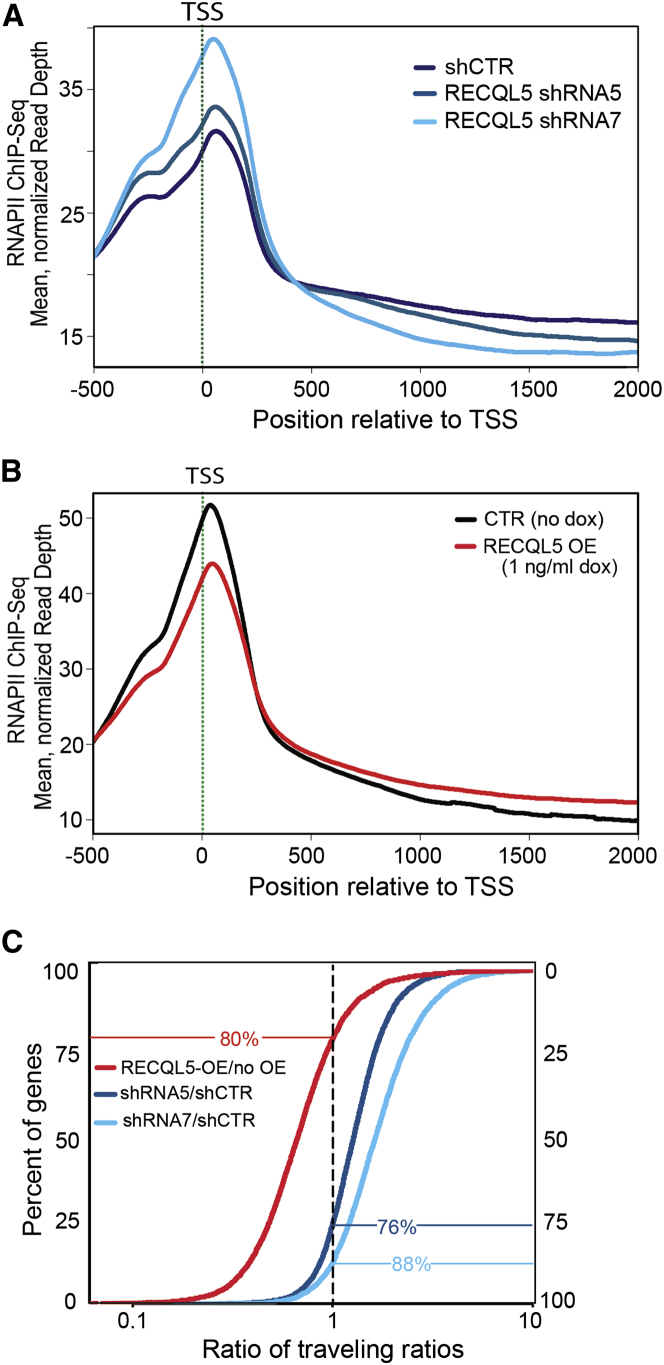
RECQL5 Depletion Changes the Profile of RNAPII across the Genome (A) Effect of RECQL5 knockdown with two different shRNAs on the RNAPII ChIP-seq profile. TSS, transcription start site. CTR, control. (B) As in (A), but after overexpression (OE) of RECQL5 (see Figure S1D). (C) RNAPII ratio of traveling ratios for 5,140 genes after knockdown or overexpression of RECQL5, relative to that in the control (set to 1). y axes indicate percent of all genes. See also Figure S1 and Table S1.

**Figure S1:**
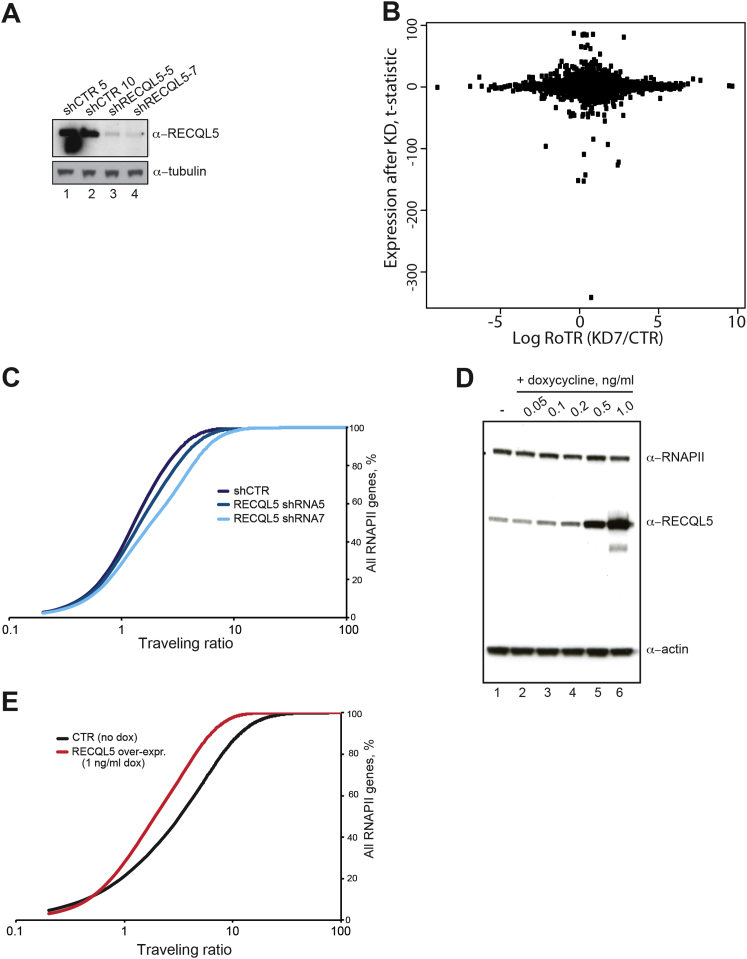
RECQL5 Levels Affect RNAPII Profiles Genome-wide, but Have Little Effect on Gene Expression Levels, Related to Figures 1 and 2 (A) Western Blot analysis showing the efficiency of the two shRNAs against RECQL5, with tubulin as loading control. Both experimental shRNAs knock down RECQL5 efficiently, but shRNA7 consistently repeatedly worked better than shRNA5 (lanes 3 and 4). Note that shCTR5 and shCTR10 are two doses of viral supernatant of the control (CTR) shRNA, showing that the infection procedure itself does not affect RECQL5 protein levels (lanes 1 and 2). (B) Correlation plot, between gene-specific changes in the RNAPII profile (x axis; ratio of traveling ratios, RoTR), and changes in the expression of these genes (y axis; t-statistic of expression changes) upon RECQL5 knockdown. As suggested by this plot, there is little change in expression values between wild-type and knockdown (y axis). See also Table S1. More importantly, there is no correlation between genes whose expression change and those at which there is a change in promoter-proximal peak density (relative to density in coding sequence; traveling ratio) (p value > 0.7; and Spearman’s correlation −0.05). (C) RECQL5 knockdown induces a shift in the traveling ratio. Traveling ratios were calculated for all the Ensembl transcripts and plotted, sorted according to their values. The plots show that upon RECQL5 knockdown the traveling ratios generally shift toward higher values, supporting the finding that the increased RNAPII density on the TSS *and* the decreased density of RNAPII in the body of the gene is a general feature. (D) Western Blot showing RECQL5 levels with increasing doses of doxycycline. Cells were treated for 24h at the indicated concentrations of doxycycline before being harvested. RNAPII and actin are used as loading controls. (E) As in (C), but after overexpression of RECQL5, showing that the traveling ratios generally shift toward lower values, supporting the finding that the decreased RNAPII density on the TSS *and* the increased density of RNAPII in the body of the gene is a general feature.

Previous experiments indicated that RECQL5 can inhibit transcription, and transcript elongation in particular, in reactions reconstituted with purified transcription proteins (Aygün et al., 2009). To investigate its role in vivo, we performed RNAPII ChIP-seq analysis after RECQL5 depletion. RECQL5 knockdown was achieved using two different lentivirus-driven shRNAs that substantially reduced protein levels, with higher knockdown efficiency reproducibly achieved using shRNA7 than shRNA5 (Figure S1A available online). Upon RECQL5 depletion, a striking genome-wide increase in RNAPII levels over transcription start sites (TSS) was observed, with the increase in peak size correlated with knockdown level (Figure 1A). However, RECQL5 depletion had little effect on total mRNA levels, and there was no correlation between the effect of RECQL5 depletion on promoter-proximal peaks and gene expression (Spearman correlation coefficient −0.05; see Figure S1B and Table S1 for expression data), indicating that RECQL5 does not significantly affect transcription initiation frequency.

Intriguingly, an inversion of the relative RNAPII levels across genes was observed approximately 500 bp downstream from the TSS, with less RNAPII being observed in transcribed regions upon RECQL5 knockdown. This effect was again reproducibly more severe with increasing knockdown efficiency, with the highest level of RECQL5 knockdown resulting in a ∼25% decrease in RNAPII density in transcribed regions (Figure 1A).

To further analyze quantitatively how RECQL5 affects transcription at the genomic level, the density of RNAPII in the promoter-proximal region relative to the gene body, previously termed the traveling ratio (Reppas et al., 2006), was calculated. In accordance with our observations, the traveling ratio was globally higher in cells lacking RECQL5 compared to control cells, again with a greater effect in cells with less RECQL5 protein (Figure S1C).

We note that RECQL5 chromatin immuno-precipitation (ChIP) and ChIP-seq analysis was also attempted, but that sites of RECQL5 occupancy could not be reproducibly detected, possibly because RECQL5 is lowly expressed and represents a “moving target” without appreciable DNA sequence preference.

### Overexpression of RECQL5 Has the Opposite Effect of Its Depletion

We repeated the RNAPII ChIP-seq experiments, but this time after overexpressing RECQL5 (see Figure S1D). Gratifyingly, the effect of RECQL5 overexpression on RNAPII distribution was the opposite of that observed upon knockdown: it reduced RNAPII density over the promoter and TSS and resulted in increased levels across the transcribed regions (Figure 1B). In further support of a genome-wide effect of RECQL5 overexpression, the traveling ratio moved toward lower values under these conditions (Figure S1E).

The traveling ratios given above were for all genes, whether they presented a clear promoter-proximal RNAPII peak or not. Given that genes with a promoter-proximal peak are more likely to be active, we next focused on 5,140 genes with clear promoter-proximal peaks, and then asked how RECQL5 affects the traveling ratio in this group of genes. Eighty percent (4,092 of the 5,140 genes) had a decreased traveling ratio upon RECQL5 overexpression. Conversely, 76% and 88% had an increased traveling ratio upon RECQL5 knockdown with shRNA5 and shRNA7, respectively (Figure 1C). Importantly, there was a substantial overlap between the genes that responded to either loss or increase of RECQL5 expression: among the 4,092 genes whose traveling ratio decreased upon RECQL5 overexpression, 3,641 (89%) were affected in the opposite direction by RECQL5 knockdown with shRNA7 (p value for overlap: 2.08 × 10^−9^). Thus, the same set of genes responded to a change in RECQL5 levels.

### RECQL5 Decreases the Elongation Rate of RNAPII

**Figure 2:**
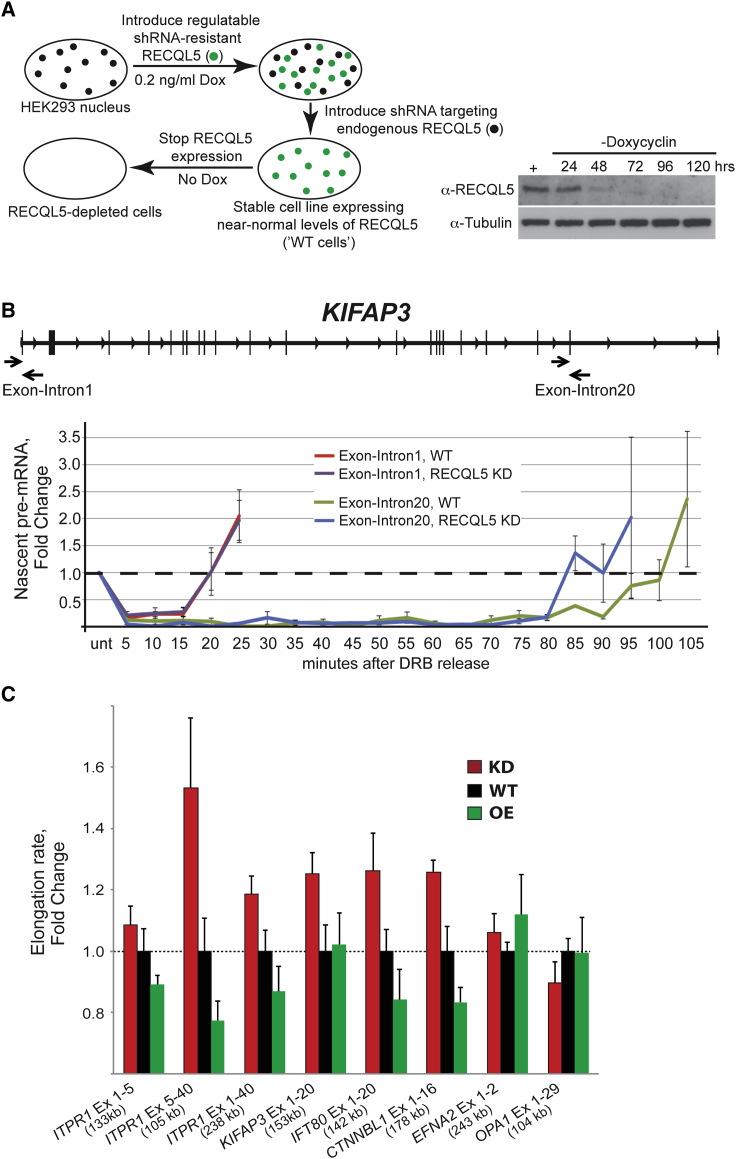
RECQL5 Inhibits Transcript Elongation at a Number of Long Human Genes (A) A system for regulating RECQL5 expression in cells lacking the endogenous protein. (B) Nascent mRNA production in different regions of the *KIFAP3* gene after release from DRB-inhibition. (C) As in (B), but in different areas of different long genes, after either reducing (KD, red), or increasing (OE, green) the cellular level of RECQL5. Average values of a minimum of three independent experiments, with standard errors, are plotted.

We next tested whether RECQL5 might play a role in RNAPII transcript elongation in vivo. In order to first establish an effective and specific system for regulating RECQL5 levels, we created a human cell line in which an shRNA-resistant version of RECQL5 was expressed under the control of a doxycycline-inducible promoter. This allowed stable shRNA knockdown of the endogenous version of RECQL5. In this “RECQL5-shutoff” cell line, RECQL5 is expressed at near-normal levels, but doxycycline removal results in its efficient depletion (Figure 2A). Because the levels of RECQL5 are controlled only by doxycycline, this system effectively controls for off-target effects of shRNA treatment. This cell line was used in all subsequent in vivo experiments.

Singh and Padgett (2009) previously used 5,6-dichlorobenzimidazole 1-beta-D ribofuranoside (DRB) to measure RNAPII transcript elongation rates in vivo. DRB inhibits P-TEFb–dependent phosphorylation of Spt5 and Serine 2 of RNAPII’s C-terminal domain (CTD), resulting in a failure of newly initiated RNAPII to progress to the elongation phase while permitting mature elongation complexes to complete transcription. DRB thus reversibly blocks new transcript elongation and in effect synchronizes the transcription cycle. Polymerases can then be concomitantly released by inhibitor removal, so that the time-resolved arrival of RNAPII at different intron-exon junctions can be assessed. Figure 2B shows how transcription through the *KIFAP3* gene proceeded after DRB removal. As expected, transcription over the first, promoter-proximal exon-intron junction was indistinguishable between the two cell lines (red and purple graphs). In contrast, pre-mRNA levels at an exon-intron junction 153 kilobases (kb) downstream from the TSS rose markedly earlier in the cell line depleted for RECQL5 (10–15 min; compare blue and green graphs). This corresponds to an overall mean elongation rate across the *KIFAP3* gene of 2.34 kb/min in the knockdown compared to 1.87 kb/min in the WT (i.e., a 25% rate increase in the absence of RECQL5).

We similarly compared elongation rates over seven regions in long human genes, and observed an increased transcript elongation rate at most, but not all of them, with an increase of ∼20% on average (Figure 2C, compare red and black bars). Importantly, overexpression of RECQL5 gave rise to the opposite effect: it generally reduced elongation rates (compare green and black bars). The decreases were consistently more severe at genes in which greater increases were observed upon RECQL5 depletion (e.g., *ITPR*ex5-40 and *IFT80*), suggesting that elongation in these regions is particularly sensitive to RECQL5 levels. Conversely, regions in which little or no change in elongation rates was observed upon RECQL5 depletion were also largely unaffected by overexpression (e.g., *EFNA2* and *OPA1*). These results are in line with those obtained by measuring traveling ratios: the same sets of genes are affected by RECQL5 depletion and overexpression. The results are also in agreement with the RNAPII ChIP-seq results above, as faster transcript elongation will result in lower RNAPII density in the transcribed region of genes (Ehrensberger et al., 2013).

### DRB/GRO-Seq Measures RNAPII Elongation Rates across Genes, Genome-wide

**Figure 3:**
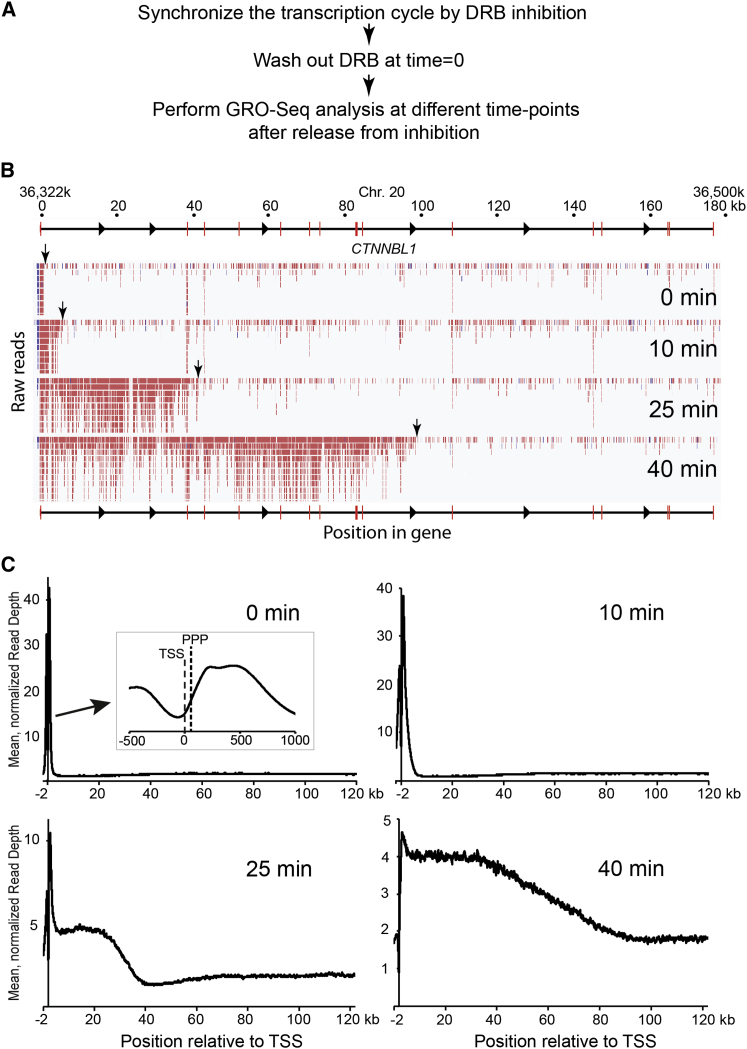
DRB/GRO-Seq to Measure RNAPII Progress across Genes, Genome-wide (A) Schematic of DRB/GRO-seq. (B) GRO-seq reads of nascent RNA produced at the *CTNNBL1* gene at different times after release from DRB-inhibition of transcript elongation. (C) Profile of normalized GRO-seq reads across 8,529 genes in the human genome over time. The insert represents an enlargement of the area close to the TSS. PPP, promoter-proximal RNAPII peak, observed by ChIP-seq.

We next used DRB in combination with GRO-seq analysis (Core et al., 2008), hereafter called DRB/GRO-seq, as a new method to analyze RNAPII elongation rates genome-wide. In DRB/GRO-seq, the position of RNAPII in the body of genes is analyzed by extending nascent RNA with BrUTP in run-on experiments performed at different times after DRB removal (Figure 3A). BrU-labeled RNA is then purified and subjected to deep sequencing. Transcription at the *CTNNBL1* gene shows the potential of the technique (Figure 3B). Prior to the release from DRB inhibition (time = 0), the vast majority of reads were observed over a narrow area near the promoter. However, as time progressed, deep sequencing reads were observed further and further into the gene (the transcription “wave front” is indicated by vertical arrows). Likewise, when assessing the data as averages across appropriately long genes (8,529 genes, >30 kb), RNAPII activity was observed at the beginning of genes at time = 0, 200–500 nucleotides downstream from the TSS, but distinct from the promoter-proximal peak (PPP) observed by RNAPII ChIP-seq (Figure 3C, time = 0, see insert). It only moved into the body of genes upon drug removal, in line with the idea that DRB inhibits phosphorylation events required for the full function of the RNAPII elongation complex. The RNAPII wave front progressed 30–40 kb into genes after 25 min (min), while it reached 80–90 kb on average 40 min after DRB removal.

### RECQL5 Depletion Increases Transcript Elongation Rates, Genome-wide

**Figure 4:**
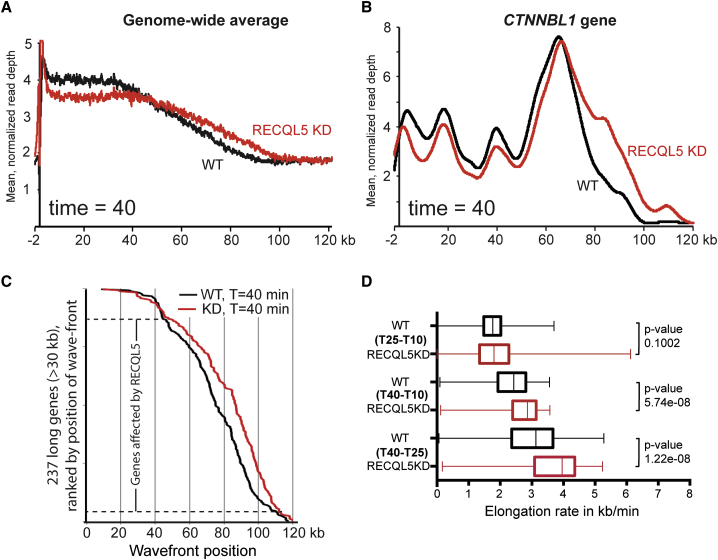
RECQL5 Loss Increases Transcript Elongation Rates across the Genome (A) Profile of normalized GRO-seq reads across the human genome 40 min after release from DRB-inhibition. (B) Normalized and smoothed spline profile across the *CTNNBL1* gene. The read-depth differs across the gene, giving rise to apparently uneven activity distribution. (C) Positions of RNAPII wave fronts calculated over appropriately long genes 40 min after DRB removal. p value for differences between data sets <10^−4^. (D) Transcription elongation rates in the indicated time intervals calculated from the position of RNAPII activity wave fronts. All genes included had to be long enough for transcript elongation to not have reached the TTS at the time of measurement, so fewer genes could be tested at later time points (see Extended Experimental Procedures). Box-plot representation shows median values ± 25% quartiles in the box and minimum/maximum distribution of the values in the whiskers. See also Figure S2.

**Figure S2:**
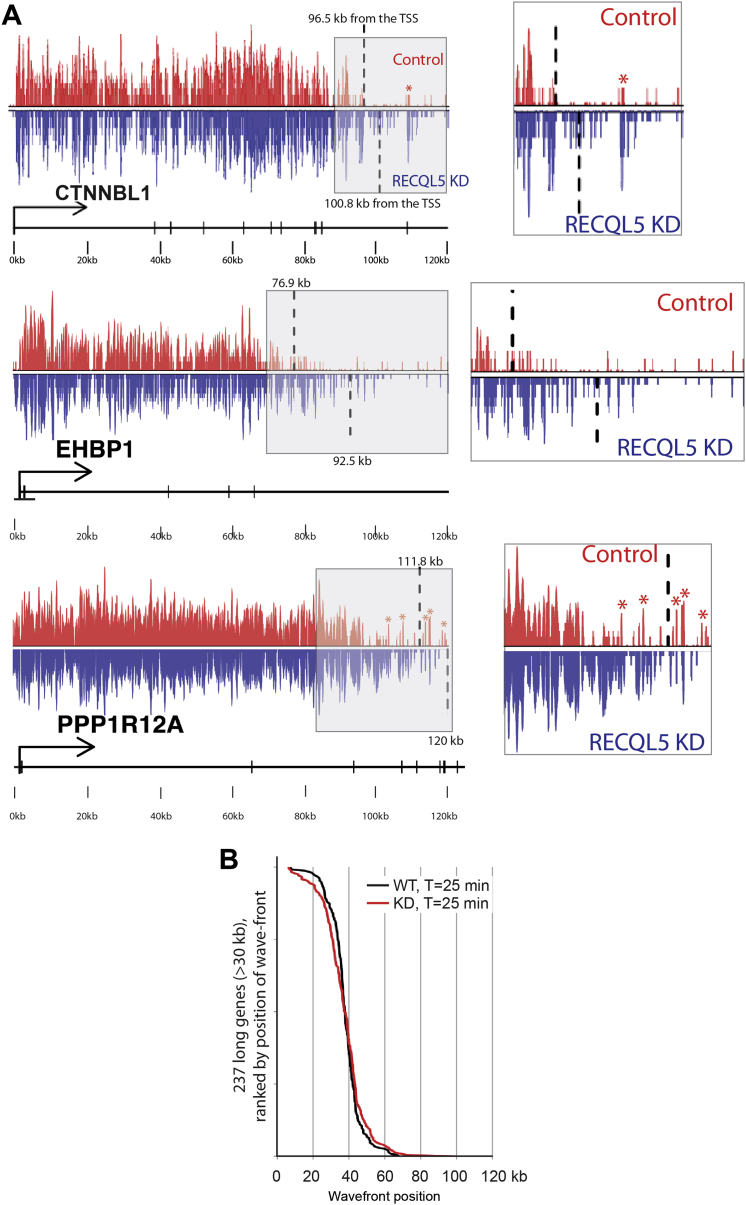
RECQL5 Inhibits Transcription Elongation, Related to Figure 4 (A) Individual examples of DRB/GRO-seq traces before and after RECQL5 depletion, with computer-called wave fronts indicated (stippled lines; gray-shaded areas enlarged on right). Note that exons, presumably because of contamination with mRNA, often give a background signal (red asterisks above WT trace; refer to gene schematic below [exons as vertical bars]), which is taken into account by the wave front-calling protocol. (B) Positions of RNAPII wave fronts calculated over appropriately long genes 25’ after DRB removal. No marked difference in the position of the wave fronts is observed in the RECQL5 KD cells compared to the control.

Having established that DRB/GRO-seq can be used to characterize transcript elongation in vivo, we now compared wild-type cells with cells lacking RECQL5 (Figure 4). Significant differences were difficult to detect at the early time points. After 40 min, however, the RNAPII-activity wave-shape was clearly altered in the RECQL5 knockdown cells, with relative depletion of polymerases in the area up to around 40 kb and with a corresponding density increase in the region from 40 kb to the transcription wave front at ∼100 kb (Figure 4A), indicating that RNAPII generally transcribed further into genes in the absence of RECQL5. Figure 4B shows a specific example in which more nascent RNAPII transcript reads were detected 80–120 kb into the *CTNNNBL1* gene in the absence of RECQL5 than in normal cells (more examples in Figure S2A).

We also determined the positions of the RNAPII wave fronts at 10, 25, and 40 min in appropriately long genes (Figures 4C and S2B, and data not shown). These were selected only on size and other genomic parameters, and not on differential behavior in the two cell types, but RECQL5 affected transcript elongation rates at the majority of them. Indeed, with elongation rates varying dramatically from gene to gene, only those at which transcript elongation was very slow, or extremely fast, were unaffected by RECQL5 depletion (Figure 4C). Moreover, using the wave front positions, the median elongation rates could be calculated (Figure 4D). In keeping with recent results (Danko et al., 2013), transcript elongation rates close to the promoter (i.e., at the early time points) were markedly lower than during elongation further into the gene (25–40 min comparison at bottom). This also helps explain the difference between elongation rates obtained by qPCR (Figure 2C) and DRB/GRO-seq. More importantly, elongation rates were significantly higher in cells lacking RECQL5: the median elongation rate in the 25–40 min interval was 3.96 kb/min in RECQL5-depleted cells and 3.13 kb/min in wild-type cells, a rate increase of 27% in the absence of RECQL5 (p value 1.22 × 10^−8^) (Figure 4D). Moreover, among the randomly selected genes in which elongation rates could be confidently measured, 77.7% had increased elongation rates in the mutant in the 25–40 min interval (and 78.4% in the 10–40 min interval). Together, these experiments indicate that RECQL5 moderates transcript elongation rates genome-wide.

### Chromosomal Rearrangements in Cells Lacking RECQL5

**Figure 5:**
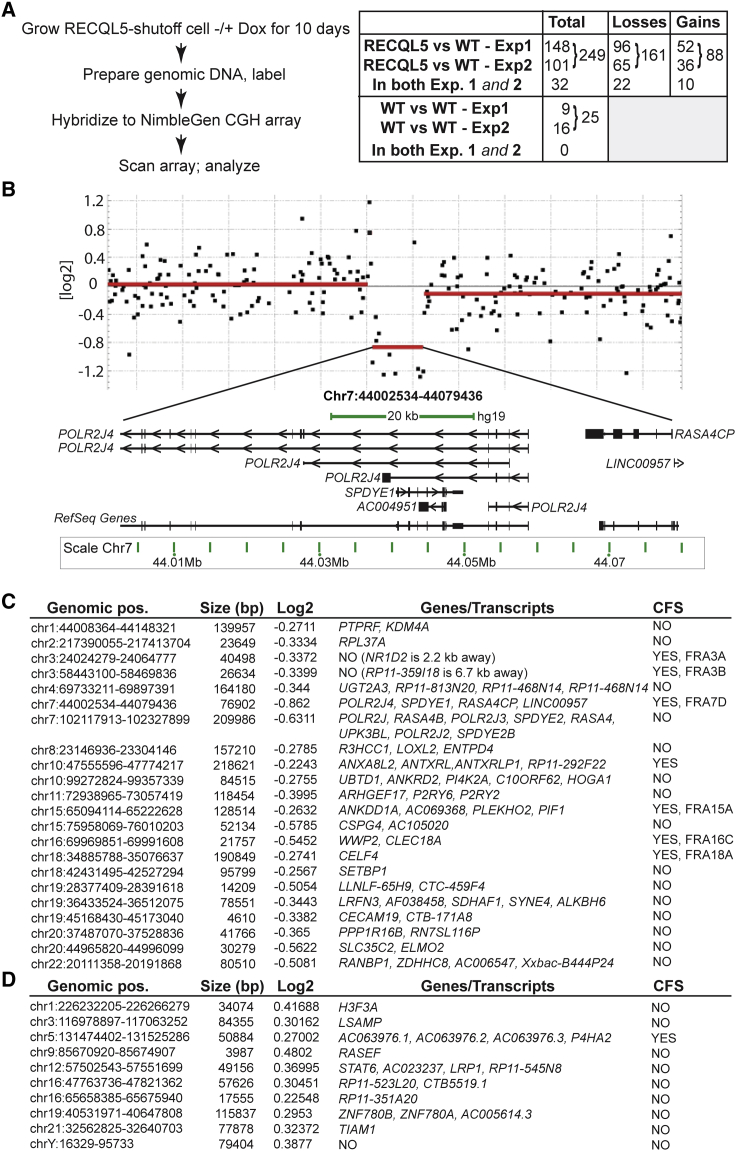
RECQL5 Depletion Results in Chromosomal Instability (A) Left: outline of CGH approach. Right: gains or losses of genomic regions upon RECQL5 depletion and in WT cells. (B) Example of gene-rich region on chromosome 7p13, lost upon RECQL5 depletion. Each black dot represents a different probe on the microarray and the red lines their mean. Expanded area depicts only some of the annotated transcripts in the lost region. (C) Recurrent lost regions and their correlation with transcription, as well as CFSs. (D) As in (C), but for regions of gain. See also Tables S2, S3, and S4.

Previous results implicated RECQL5 in maintaining genome stability, but whether this role is connected to the effects on transcript elongation established here was unclear. We used comparative genomic hybridization (CGH) to investigate whether chromosomal rearrangements occur upon RECQL5 depletion, and, if so, whether such rearrangements were associated with transcription. CGH compares two genomic DNA samples to detect differences such as gains or losses of whole chromosomes or, more likely, chromosomal regions (Park, 2008). A batch of RECQL5-shutoff cells grown in the presence of doxycycline was split in two and grown either in the continued presence of doxycycline (to maintain RECQL5 expression [“wild-type”]), or without it to deplete RECQL5. Genomic DNA from the two cell populations was then compared (Figure 5A, left; and 5B). Because these cells were identical at the outset, any change in copy number will have occurred during this short period of growth (10 days, typically 10–14 cell divisions). Strikingly, no less than 249 genomic losses and gains occurred in response to RECQL5 depletion in the two independent comparisons, using a log2 ratio threshold of 0.2 (Figure 5A, right). This threshold signifies that more than ∼15% of cells in the RECQL5-depleted cell population carry the particular rearrangement and therefore provides a conservative measure of the total number of genomic rearrangements taking place. The large number of genomic alterations (161 losses and 88 gains; see also Table S2) inspired us to check the basal level of genome instability in the wild-type cell line itself. This is relevant because in CGH, one of the samples (the wild-type) is defined as unchanging, and genomic rearrangements in the test sample are then described relative to this reference. Again, the cell culture was split in two, but with RECQL5 expression maintained in both, before the genomes of the clones were compared. In this CGH comparison, gains and losses cannot be distinguished, as the reference sample is arbitrarily set (e.g., a loss detected in the “test” sample might equally well be a gain in the “reference” sample). A total of 25 genomic alterations were detected in two independent experiments (16 and 9, respectively), 10-fold less than observed when RECQL5 was depleted (Figure 5A, right; Table S3).

Interestingly, although the size of the rearrangements observed upon RECQL5 depletion was relatively small (median was 45 kb for losses, and 37 kb for chromosomal gains), we identified no less than 22 examples of loss (out of 65 and 96, respectively) and 10 gains (out of 36/52) that occurred in both independent experiments (Figures 5A, right, 5C, and 5D). So, around a third of the genomic rearrangements in one experiment were also detected in the other. It is obvious that the likelihood of such repetition happening by chance is miniscule. Indeed, the p value can be estimated by a permutation test and is <3 × 10^−4^. In contrast, none of the rearrangements detected when comparing WT with WT were recurring, and none of these unique events overlapped with the recurring RECQL5-related events.

We surmised that the recurring genomic rearrangements (22 examples of loss, 10 examples of gains; Figures 5A, right; see also Figures 5C and 5D) could be key to understanding RECQL5 function, so we initially focused on these high-confidence events. When exploring common features of the regions affected, we noted that almost a third (7 of 22; p value 0.033) of the loss regions reside inside one of 124 replication-dependent, common fragile sites (CFSs) mapped in human autosomes (Fungtammasan et al., 2012). Replication programs are cell-type-specific (Letessier et al., 2011), and CFSs have not been extensively mapped in HEK293 cells. The overlap of RECQL5-dependent genomic losses was thus with CFSs mapped in other cell types (typically human lymphocytes), making it all the more noteworthy. No statistically significant overlap was observed between CFSs and RECQL5-related gains (p value 0.319) or the events observed in wild-type cells (p value 0.217).

We also checked whether RECQL5-related genomic instability was associated with transcription. Figure 5B shows an example. This region (Chr7p13) is indeed gene-rich and positioned inside CFS FRA7D. Regions of recurring genomic loss generally contained genes or annotated transcripts: 20 of the 22 recurring losses encoded at least one RNAPII transcript, and the remaining two were within a few kilobases of a gene (Figure 5C; Table S2). Moreover, genomic losses not only overlapped with transcription, but either one or both of their breakpoints were within an annotated transcript in 82% of cases (p value 4.87 × 10^−5^). No statistically significant overlap with genes was observed with the breakpoints of recurring genomic gains (p value 0.9997) (see also Figure 5D), or with rearrangements in wild-type cells (p value 0.621).

We now analyzed the entire set of RECQL5-dependent genomic alterations (not only recurring events) to establish whether the associations were generally observed. Gratifyingly, the 161 RECQL5-related genomic losses were generally associated with CFSs and genes: 36 events (22%) overlapped with CFSs (p(permutation) 0.048), and 87% overlapped with genes (p(permutation) < 10^−4^). In contrast, no statistically significant association was observed with RECQL5-dependent genomic gains, or with rearrangements observed in wild-type cells (p(permutation) 0.420 and 0.217, respectively, for CFSs; and p value 0.902 and 0.171, respectively, for genes). Again, either one or both of the breakpoints of the genomic losses were typically within the transcribed region of an annotated gene or transcript (in 80% of cases; p value 3.21 × 10^−11^), while no significant general association was observed for genomic gains, or for the wild-type (p values of 0.902 and 0.621, respectively). We failed to detect overlap between RECQL5-dependent genome instability and so-called early replicating fragile sites (ERFSs) (p values 0.162 and 0.585, respectively, for losses and gains), with the caveat that these ERFSs were characterized in the mouse (Barlow et al., 2013), so that our comparisons could only be made in a limited number of syntenic regions.

Together, these data show that RECQL5 depletion gives rise to genome instability associated with common fragile sites and the transcribed region of genes.

### Characteristics of Genes Containing Sites of RECQL5-Dependent Genome Instability

We now investigated the possible relationships between transcript elongation and genome instability, focusing primarily on genomic loss-associated chromosomal breaks as these were correlated with genes and CFSs. All in all, 164 well-defined genes or long noncoding transcripts with a total of 212 genomic loss-associated breakpoints in their transcribed region were suitable for such examination.

Bioinformatic analysis showed that genes with genomic loss-associated chromosomal breaks in their coding regions were inconspicuous with regard to their gene expression level (i.e., they were neither particularly highly nor lowly expressed genes), and their change in expression upon RECQL5 depletion (i.e., they did not represent a sub-group whose expression was particularly RECQL5-dependent). Moreover, the breakages observed were at varying distances from the TSS and TTS, and not, for example, enriched at either end (see Table S4 for information on individual break sites in genes).

We noted that there was a tendency for genes with loss-associated chromosomal breaks in their coding regions to be long, with the mean length of such genes being 123 kb, compared to 58 kb for all human genes (median length was 74 kb, compared to 23 kb for all human genes). As an aside, although the RECQL5-dependent genomic gains were not associated with genes to a statistically significant degree (see section on CGH above), either one or both gain-associated chromosomal breakpoints were nevertheless inside the transcribed region of a gene in no less than 80% of cases. These genes were typically very long, with a median length of 105 kb and a mean length of 254 kb; indeed, 23% (15 of 64) were more than 300 kb long (against ∼3.4% in the entire human genome). However, all else being equal, long genes would also be more likely to be hit be a damage-event occurring at random, and although the enrichment of long genes might well be biologically meaningful, it was not statistically significant.

Most importantly, however, we were unable to find a connection between elongation speed and chromosomal breakage. In both loss- and gain-associated breakage genes where it could be estimated or calculated via DRB/GRO-seq, the elongation rate was thus close to the median rate established for all measurable genes in Figure 4 (i.e., their rate did not change in a way that distinguished them in the background of changes that occurred when RECQL5 is lost). In agreement with this, the traveling ratios of genes with chromosomal breaks were also unremarkable compared to control genes without breaks. Together, these data show that although genes with RECQL5-dependent chromosomal breaks in their coding region may be long, they do not stand out as unusual by a number of other parameters compared to the much larger group of control genes that did not display genome instability.

### RECQL5-Related Transcription Stress

The absence of a clear connection between genome instability and elongation rates prompted us to investigate the role of RECQL5 in transcript elongation in more detail. Besides providing an overview of polymerase density across genes genome-wide, RNAPII ChIP-seq also uncovers local areas of increased density (peaks) within individual genes. RNAPII peaks are typically broad, often comprising several kilobases of DNA, with those in the transcribed region of genes indicating areas of increased pausing or arrest (i.e., transcription stress) (Ehrensberger et al., 2013). Somewhat unexpectedly, individual RNAPII peak-calling using MACS (Zhang et al., 2008) uncovered a marked increase in the number of individual peaks with increasing RECQL5 knockdown (2.4- and 3.2-fold more after shRNA5 and shRNA7 knockdown, respectively). Given that RECQL5 depletion results in higher promoter-proximal peaks (Figure 1), a trivial explanation for these increases could be that more promoters start to display detectable promoter-proximal peaks upon RECQL5 loss. We therefore examined the 5,140 genes that already had a promoter-proximal RNAPII peak. Interestingly, instead of calling a decreased number of RNAPII ChIP-seq peaks in these genes as might have been expected from the metagene-analysis (Figures 1A and 1C), MACS called a markedly *in*creased number of individual gene body peaks upon RECQL5-depletion (4.0- and 5.2-fold more after shRNA5 and shRNA7 knockdown, respectively; p values both <10^−4^). These data suggest that although RNAPII on average traverses genes more rapidly in the absence of RECQL5, this is accompanied by incidents during which polymerase is paused or arrested in the transcribed region of genes.

**Figure 6:**
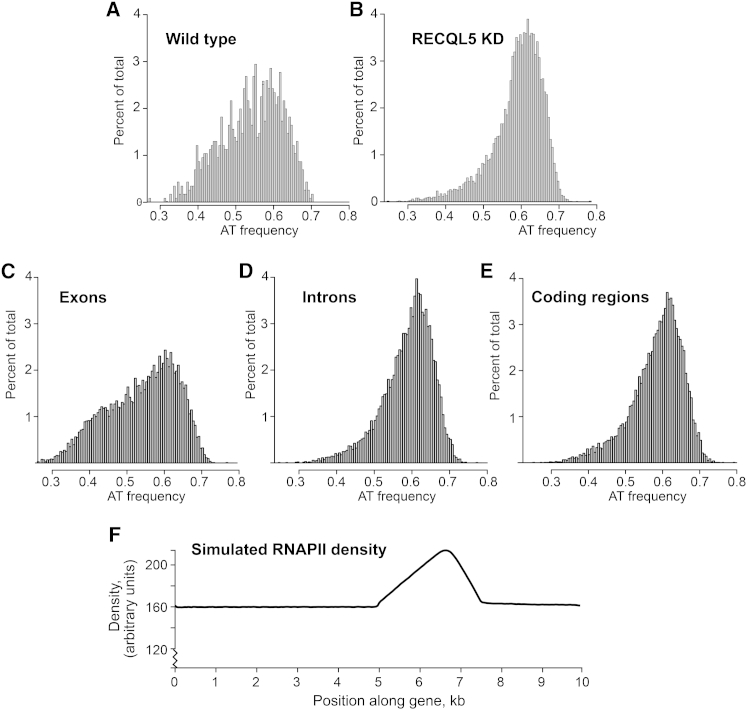
RECQL5 Depletion Results in Transcription Stress across the Transcribed Region (A–E) Mean AT frequency distribution of ±1 kb regions around RNAPII peaks in (A) wild-type, (B) RECQL5 knockdown cells (shRNA7), (C) exons, (D) introns, and (E) transcribed regions overall. See also Figure S3. (F) CHIPMOD simulation of stochastic RNAPII pausing by 1% of the polymerase population. Parameters used, other than default: 9.99 kb gene; pause start at 5 kb; width 2.5 kb; 40% of normal (3.8 kb/min) elongation rate. Mean width of actual RNAPII ChIP-seq peaks is 2.7 kb; see Figure S4 for examples.

**Figure S3:**
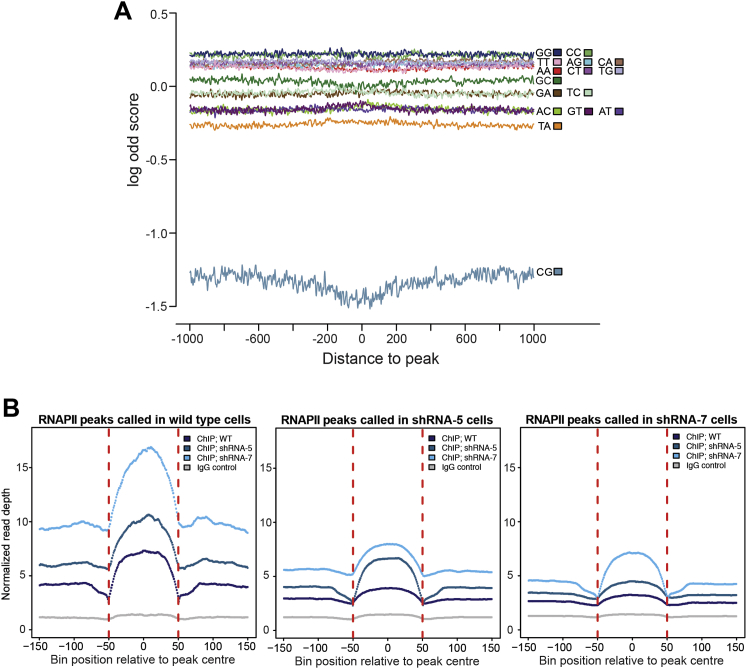
RNAPII Peaks; Sequence and Enrichment Characteristics, Related to Figure 5 (A) Di-nucleotides frequencies around RNAPII peaks can be explained by mononucleotide frequecies profiles. The y axis shows position specific log-odds ratios between the di-nucleotide frequencies and their expected frequencies based on the mononucleotide frequency profiles. Similar results were seen at greater distances from RNAPII peaks. As expected, CpG di-nucleotides are under-represented in transcribed regions. (B) RNAPII ChIP-seq peaks in wild-type, shRNA5-, and shRNA7-treated cells overlap. The average, local RNAPII density over peaks called by MACS in the different experiments was calculated in the other experiments as indicated (see color coding). Note that peaks called in wild-type cells (top) on average also had a clear, local increase in RNAPII density in the area upon shRNA5- and shRNA7-mediated RECQL5 depletion. Conversely, peaks called in cells depleted for RECQL5 by shRNA5 (middle) and shRNA7 (bottom) also correlated with an average local increase in RNAPII density in wild-type cells, albeit to a much more modest extent. Method: Each peak region was split into 100 equally sized genomic intervals (bins). The 2 kb region either side of each peak were also split into 100 intervals, together making for 300 genomic intervals centered around the midpoint of each peak. The mean read depth in each bin was calculated for all transcripts and a final mean across all bins was plotted. The red dotted lines delineate the boundaries of the peaks, so from this point of view the x axis is perhaps a little misleading - everything from −150 to −50 is upstream of the peaks, −50 to +50 is the peak itself and +50 to +150 is the region downstream of the peak.

To gain further insight into the nature of the intragenic RNAPII peaks, we aligned the underlying sequences in the hope of uncovering similarities. Neither sequence consensus elements, nor strongly over- or under-represented oligonucleotides given the background frequencies were uncovered (Figure S3A; data not shown). However, regions affected by RECQL5 depletion had a distinct mononucleotide frequency distribution, with the peaks in RECQL5-depleted cells generally being more AT-rich than those in wild-type cells (Figure 6, compare A and B). In general, exons are significantly more GC-rich than introns and also—because introns are typically 10-fold longer than exons – than transcribed regions in general (Louie et al., 2003) (Figures 6C–6E). In apparent agreement with the finding that RNAPII tends to pause at intron-exon junctions (Kwak et al., 2013), the sequence-signature of RNAPII-peaks in wild-type cells looked more similar to that of exons than introns (Figure 6, compare A with C and D). In contrast, RNAPII peaks in RECQL5-depleted cells had an AT-signature more similar to that of introns or transcribed regions in general (Figure 6, compare B with C, D and E), suggesting that the increased transcription stress observed upon loss of RECQL5 is distributed across transcribed regions.

It is worth noting that genomic areas containing an RNAPII peak in the wild-type cells also typically harbored increased RNAPII density after RECQL5 depletion. The opposite was less obvious, but still noticeable (Figure S3B). The much higher frequency of detectable RNAPII pausing and arrest in RECQL5-depleted cell therefore does not imply that transcription stress *only* occurs in RECQL5-depleted cells, nor that RNAPII *only* pauses at intron-exon junctions in wild-type cells. Rather, it suggests that while the same causes underlie pausing and arrest in RECQL5-depleted cells, wild-type cells can better buffer impediments to elongation.

It may appear counter-intuitive that RECQL5 depletion results in both elevated transcription stress *and* faster transcript elongation. However, computer modeling by an updated version of CHIPMOD (Ehrensberger et al., 2013), which can now also simulate stochastic and rare polymerase behavior, supports the idea that transcription stress can be detected even if such events are very rare. Indeed, even if merely 1% percent of transcribing polymerases experience modest pausing through, or transcriptional arrest in, a gene region, this has a striking effect on the predicted RNAPII density profile (Figure 6F, data not shown).

Together, these results suggest that RECQL5-depleted cells suffer from substantial transcription stress, so that although elongation rates are higher, this is accompanied by a more interrupted transcript elongation process: events during which elongating RNAPIIs stochastically pause or arrest.

### Evidence that RECQL5-Related Transcription Stress Causes Genome Instability

**Figure S4:**
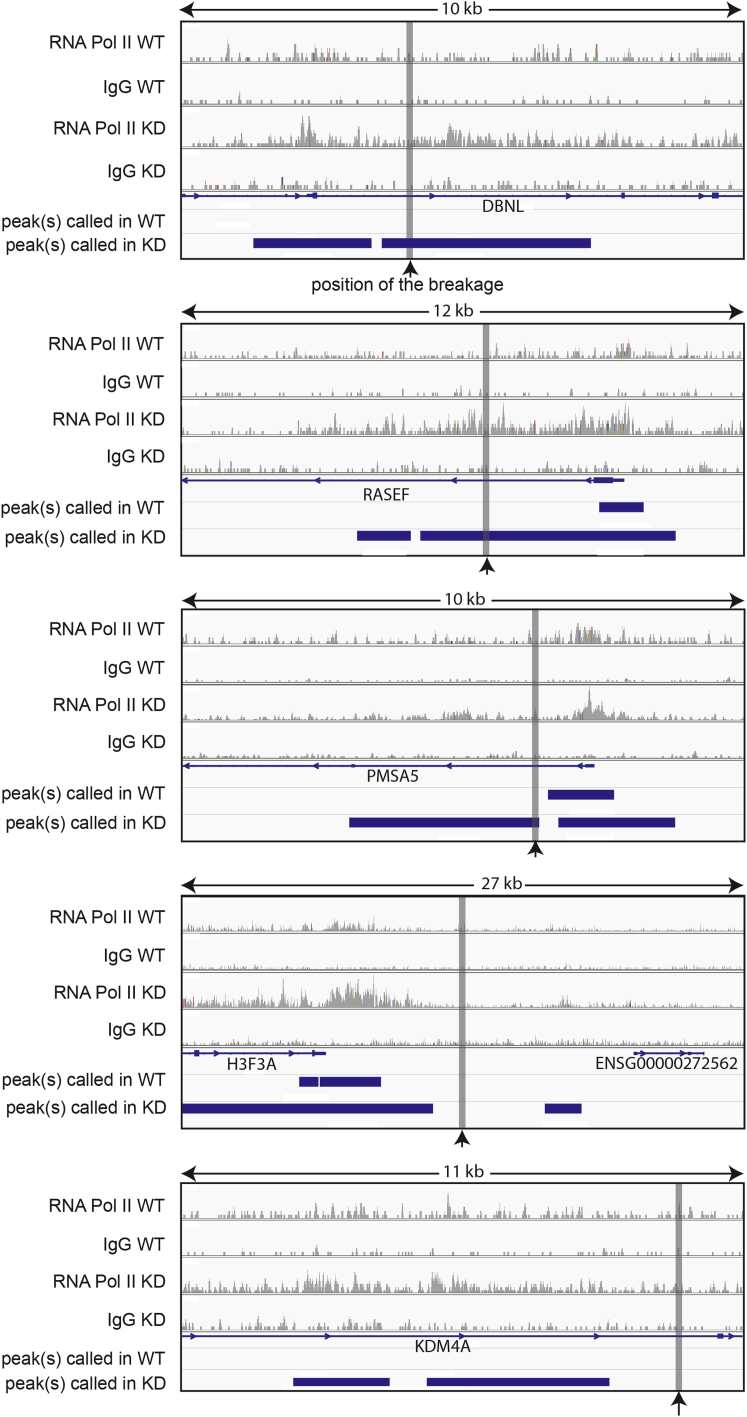
Examples of RNAPII Peaks near Sites of Chromosomal Breakage, Related to Figures 5, 6, and 7 Blue horizontal lines indicate individual RNAPII peaks called by MACS (Zhang et al., 2008), while vertical gray lines indicate chromosomal breakpoints called by the CGH software. For simplicity, only read traces from WT (WT), shRNA7 knockdown (KD), and their associated IgG controls (IgG) are shown. Genes and their direction of transcription are also indicated. Note that the break in the example including the H3F3A gene (second from bottom) is not inside a gene, but is located between two RNAPII peaks, one of which is not in an annotated transcript.

We finally investigated whether RECQL5-dependent genome instability might be connected to transcription stress. In an ideal, imaginary experiment, transcription stress would be measured at positions of chromosomal gene breakage, but immediately *prior* to these events taking place in the individual cells that react to RECQL5 depletion. This is obviously not practically feasible. We were instead restricted to comparing the position of RNAPII transcription stress events detected by ChIP-seq with the position of chromosomal breakages detected by CGH (Examples of such events are shown in Figure S4). As a control, computer-generated chromosome breaks were made, at random, across the genome (100 trials) in a quantity that yielded random breaks in genes in numbers that were similar, on average, to those observed in the actual experiments (see Extended Experimental Procedures for details).

**Figure 7:**
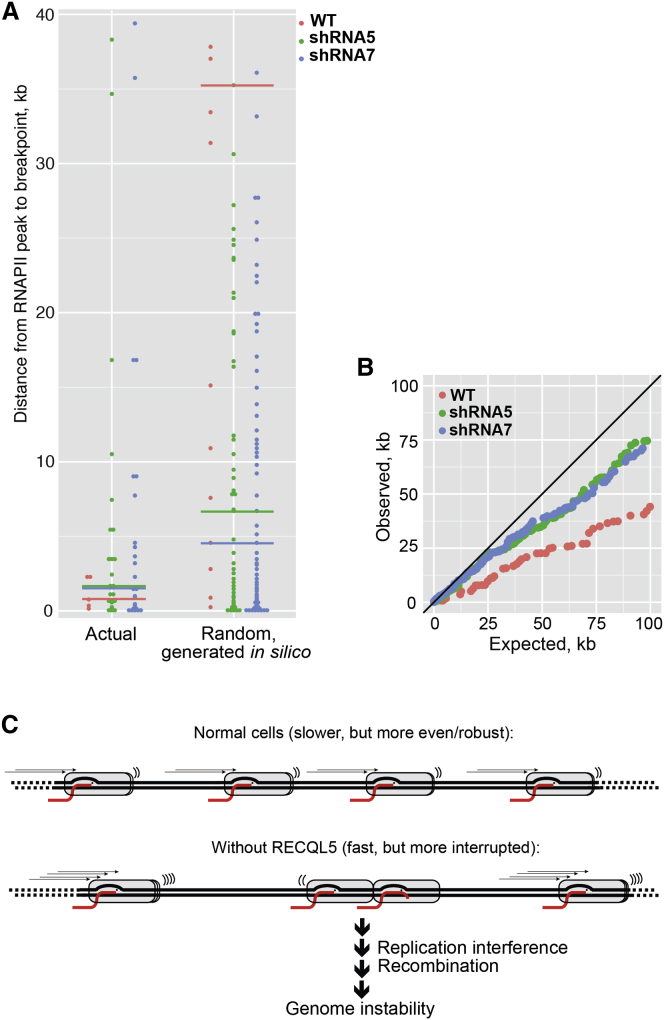
RECQL5-Dependent Genome Instability Occurs in Areas of Elevated Transcription Stress (A) Plot of distances from genomic loss-associated chromosomal breakpoints to the nearest RNAPII peak in the same gene. Left: breakpoints observed in the RECQL5 shut-off experiments. Right: simulated examples (100 independent trials, averaged by rank) of computer-generated breakpoints in genes across the human genome (as represented on the Nimblegen chip). Note that there will be fluctuation in the number of computer-generated distances due to the frequency of simulated breakpoints landing in genes with RNAPII peaks varying from simulation to simulation. Horizontal lines indicate median values (of the observed distances on the right, of the averaged randomized on the right). (B) Plot of distances from chromosomal breakpoints (observed breakpoints on y axis and random computer-generated breakpoints on x axis) to their nearest RNAPII peak, regardless of either being in a gene. (C) Model for the role of RECQL5 at the interface between transcription and the maintenance of genome stability. See text for details.

We initially focused on genomic breaks in the transcribed region of the 5,140 genes containing a clear promoter-proximal peak. Moreover, only RNAPII peaks that were in the transcribed region of the same gene as the loss-associated chromosomal break site were considered. This left 26 individual loss-associated chromosome breaks for the analysis. Remarkably, when compared to the computer-generated, random breakpoints in genes, the actual breakpoints were clearly positioned very close to the RNAPII peaks detected by ChIP-seq (Figure 7A), indicating a connection between transcription stress and genome instability in RECQL5-depleted cells.

We note that regions of transcription stress are not ever-present. Indeed, even upon RECQL5 loss, less than half of the annotated genes in the human genome had one or more RNAPII peaks called in their transcribed region. This was true whether they had breakpoints or not, and similar observations were made for genomic loss- and gain-associated breaks. When corrected for gene length (relevant since long genes would be expected to have more RNAPII peaks), genes with breakpoints actually had somewhat fewer RNAPII peaks than control genes (∼2-fold fewer), indicating that breakpoint genes were not general hotbeds of transcription stress.

**Figure S5:**
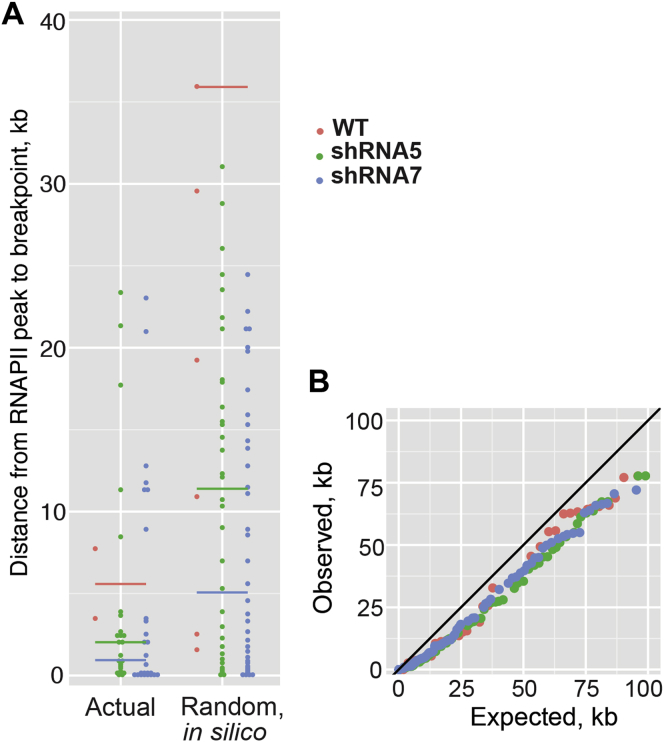
RECQL5-Dependent Genome Instability Occurs in Areas of Elevated Transcription Stress, also in the Case of Genomic Gains, Related to Figure 7 (A) Plot of distance from a genomic gain-associated chromosomal breakpoint to the nearest RNAPII peak in the same gene. The plot on the left represents distances from chromosomal breakpoints observed in the RECQL5 shut-off experiments, while the one on the right represents the distances to simulated examples (100 independent trials) of computer-generated breakpoints in genes across the human genome (as it is represented on the Nimblegen chip). In the case of randomization, the median lines are for the 100 trials of random computer-generated breakpoints. (B) Plot of distances from increasingly distant pairs of chromosomal breakpoints (observed breakpoints on y axis and random computer-generated breakpoints on x axis) to their nearest RNAPII peak. Note that whereas chromosomal breaks occurring *inside* RNAPII peak-containing test genes are clearly much closer to the RNAPII peak than would be expected by chance (S5A, compare to main Figure 7A), this is less impressive when all such breaks are investigated, irrespective of position (S5B, compare to main Figure 7B), further supporting the idea that whereas RECQL5-dependent genomic *losses* are generally strongly correlated with genes and transcription stress, RECQL5-dependent chromosomal *gains* are less strongly associated with these parameters.

We also expanded the analysis to all chromosomal loss-associated break sites and the accompanying nearest area of RNAPII transcription stress, irrespective of origin. This is relevant because RNAPII transcription is pervasive and also produces noncoding RNA, for example in enhancers and downstream from the coding region of genes. We here compiled a list of the distances between genomic loss-associated chromosomal breaks and their nearest RNAPII peak, in order of increasing distance (observed distances). A similar list was compiled for the random computer-generated chromosomal breaks (expected distances). These two distance lists were then plotted together in a scatter plot, from the shortest to the longest distance-pair (Figure 7B). In this analysis, if the distance between observed chromosomal loss-associated break sites to an RNAPII peak were no closer than would be expected by chance, the distance-pairs would lie on the diagonal line of the plot. Instead, the points were consistently below the diagonal, showing that RNAPII peaks were closer to the observed chromosomal loss-associated break sites than would be predicted by chance (Figure 7B). Intriguingly, the subgroup of genomic *gain*-associated chromosomal breakpoints that occurred inside genes were also closer to RNAPII ChIP-seq peaks than would have been expected (Figure S5), suggesting that RECQL5-dependent genome instability in genes is generally associated with areas of increased RNAPII transcription stress.

We also noted that not only the RNAPII peaks detected upon RECQL5 knockdown (Figure 7B, blue and green spheres), but also those observed in wild-type cells (red spheres) were closer to the chromosomal breakpoints than would have been expected by chance. Somewhat surprisingly, it actually appeared that the latter, much less abundant RNAPII peaks were even closer to sites of chromosomal breakage than those arising in RECQL5-depleted cells. Remarkably, however, these wild-type peaks simply represented highly consistent sites of RNAPII transcription stress, also observed after RECQL5 loss: out of the 51 RNAPII peaks plotted for wild-type cells, 49 were thus also called as peaks and plotted in either one or both the RECQL5 shRNA knockdown samples. This result is important as it implies that sites of transcription stress in wild-type cells may also give rise to genome instability. In the absence of RECQL5, however, there is both a great increase in transcription stress, and an inability to suppress the deleterious consequence for genome stability of such stress.

## Discussion

The data presented here provide evidence that RECQL5 plays a general role in the control of transcript elongation in human cells. In its absence, transcript elongation rates increase, the distribution profile of RNAPII is markedly altered across the genome, and higher levels of RNAPII pausing or arrest (i.e., transcription stress) are detected. Cells lacking RECQL5 also exhibit increased genome instability in the transcribed region of genes and at common fragile sites, and this is correlated with an increase in transcription stress in these particular regions.

### RECQL5, a General RNAPII Elongation Factor

A large number of cofactors are required for normal transcript elongation by RNAPII (reviewed by Selth et al., 2010). However, most of these factors do not appear to affect the rate of transcript elongation in vivo. For example, rate determination in yeast failed to detect an effect of factors such as Set1, Set2, Elongator, Chd1, Elongin (*ELA1)*, transcription-coupled repair factor Rad26, the Paf1 and THO complexes, Spt4, TFIIS, and Bye1 (Mason and Struhl, 2005). This in no way implies that these factors are not important for transcription, but forms a basis for appreciating the surprising discovery that RECQL5 affects transcript elongation rates at the vast majority of human genes. Moreover, using this effect as criterion, RECQL5 is a negative elongation factor: its depletion has the surprising effect of *in*creasing RNAPII elongation rate compared to wild-type cells. Nevertheless, RECQL5 does also have a significant positive effect on transcript elongation in that it decreases transcription stress and the negative effects thereof. We propose that RECQL5 may “smoothen” or “buffer” transcript elongation, resulting in a somewhat slower, but more robust and less interrupted transcription process (Figure 7C). In any case, RECQL5 represents a new type of general RNAPII elongation factor.

We note that the genome-wide analysis suggests that RECQL5 also affects not only promoter- (Figure 1), but also terminator-proximal transcription events (to be published elsewhere), but how this relates to its elongation effects remains to be investigated. It is, however, an obvious possibility that the underlying biochemical mechanism is the same.

### RECQL5 and the Maintenance of Genome Integrity

The results presented here indicate that genomic rearrangements in RECQL5-depleted cells are correlated with regions of elevated transcription stress, implying that suppression of such stress and its immediate consequence is crucial to maintain genome stability. As indicated above, it seems possible, even likely, that the involvement of RECQL5 in suppressing transcription stress (and thus maintaining genome stability) in effect comes at the cost of reducing average elongation speeds (Figure 7C).

It is worth emphasizing that transcription stress is not, in itself, genome-destabilizing. Indeed, RNAPII ChIP-seq experiments detect RNAPII pausing and arrest throughout the human genome, but this only infrequently results in genome rearrangements. Importantly, there is compelling evidence that transcription-associated genome instability requires clashes between RNAPII transcription and DNA replication (Aguilera and Gómez-González, 2008; Helmrich et al., 2013). Our results suggest that it is not transcription per se, but more specifically transcriptional pausing and arrest, which represents a challenge to genome stability during DNA replication. Recent results, albeit in bacteria (Dutta et al., 2011), support the idea that transcription stress, in this case specifically RNAP arrest and backtracking, can give rise to replication-derived genome instability.

Common fragile sites (CFSs) are genomic regions, frequently deleted in human cancer, which are predisposed to breakage when exposing cultured cells to replication stress (Debatisse et al., 2012). Recent results argue for a connection between CFSs and transcription-replication collision in long human genes (Helmrich et al., 2011; Le Tallec et al., 2013), with CFSs often situated in areas of high AT content that contain long genes (Durkin and Glover, 2007; Debatisse et al., 2012). They are also typically located in regions depleted of DNA replication origins, making replication rely on long-traveling forks, the breakdown of which cannot be compensated for by other origin firing (Letessier et al., 2011). However, the role played by transcription in CFS generation has remained unclear. Our striking finding RECQL5-dependent genomic rearrangements overlap with both CFSs and the transcribed region of long RNAPII genes points to a role for RECQL5 in suppressing breakage at CFSs. It is an obvious possibility that at least a subset of CFSs is associated with, or a direct consequence of, transcription stress. Indeed, we note that transcriptional pausing and arrest may largely be stochastic, and that it does not necessarily increase with rising gene expression levels, potentially helping to explain why the occurrence of CFSs, although typically occurring in genes, is not correlated with their transcription level (Le Tallec et al., 2013).

The biochemical mechanisms underlying DNA recombination are starting to emerge, but the possible explanations for the difference between chromosomal losses and gains remain theoretical (Arlt et al., 2012). With that in mind, we do not presently understand why RECQL5-driven genomic losses overlap much better than genomic gains with CFSs and genes. We note, however, that although the overlap between genomic gains and genes did not satisfy typical statistical confidence levels, a very large proportion of these events (80%) did occur inside genes. It is potentially important that the genes involved were typically very long and harbored regions of transcription stress near the chromosomal breakpoints, reinforcing the impression that RECQL5-dependent genomic rearrangements typically occur as a consequence of transcription-associated events. Work in chicken DT40 cells indicate that mutation of RECQL5′s RNAPII-interaction domain does not have the same severe phenotypic effect as a complete RECQL5 knockout, suggesting that RECQL5 also has a transcription-*in*dependent function in suppressing genome instability (Islam et al., 2010), which might help explain the remaining, apparently transcription-unrelated genome instability events we observed.

We note that compounds such as camptothecin and psoralen, and even endogenous DNA lesions would affect transcript elongation and result in considerable transcription stress, likely resulting in a need for the transcription function of RECQL5 described here. This might explain the intriguing connection between RECQL5 and these causes of DNA damage (see, for example, Li et al., 2011; Tadokoro et al., 2012; Ramamoorthy et al., 2013). Finally, our data potentially shed light not only on the function of RECQL5, but also on the mammalian RECQ family as a whole. These proteins evolved to enable the suppression of genome instability, including that derived from transcription-associated DNA recombination during replication. In outline, such suppression might occur by modifying the behavior of either of the proteins governing these processes, namely the replicating DNA polymerase(s), the recombination proteins, or RNA polymerase II. At this simplified, conceptual level, factors such as RECQ4 and BLM would suppress undesirable activity by DNA replication- and recombination factors, respectively (Liu, 2010; Larsen and Hickson, 2013), while RECQL5 would control elongating RNAPII, helping explain the additive or sometimes synergistic effects on genome instability levels attained when the function of RECQL5 and another RECQ gene is eliminated concomitantly (Hu et al., 2005; Popuri et al., 2013).

## Experimental Procedures

### Cell Lines

HEK293, HEK293T-Rex, and derived cell lines were grown under standard conditions. RECQL5 shRNAs were from Thermo Scientific. For generating a stable cell line in which endogenous RECQL5 was depleted so that virtually all RECQL5 was from a doxycycline-regulated gene, a RECQL5-expressing plasmid was mutagenized to make the produced mRNA shRNA-resistant. This RECQL5 form was stably expressed in HEK293T-Rex cells that were subsequently infected with lentiviral particles carrying the RECQL5 shRNA construct depleting endogenous RECQL5. Single colonies were selected with puromycin in the presence of 0.2 ng/ml of doxycycline to allow physiologic expression level of the shRNA-resistant version of RECQL5.

### Gene-Specific and Genome-wide Analysis of Transcription

RNAPII ChIP-seq was performed, with sequencing on the Illumina platform. DRB/GRO-seq integrates DRB inhibition to measure transcript elongation (Singh and Padgett, 2009) with GRO-seq (Core et al., 2008). Cells were treated with DRB for 3.5 hr, then released into DRB-free medium, with aliquots taken for GRO-seq at different time points.

### Comparative Genomic Hybridization

RECQL5 shut-off cells were grown for 10 days, either with or without doxycycline. Genomic DNA from the compared clones was isolated, labeled, hybridized to 3 × 720K human arrays, and washed according to the manufacturer’s recommendations (Nimblegen-Roche). The array was scanned by the Nimblegen MS200 micro-array scanning system. Primary data extraction, analysis, and visualization were performed using the DEVA software (Roche).

### Bioinformatic Analysis

Reads were aligned to the reference genome using Bowtie (Langmead et al., 2009) and BWA (Li and Durbin, 2009) for ChIP-seq and GRO-seq, respectively. Mapped reads were sorted and indexed using SAMtools (Li et al., 2009). RNAPII peaks were called using MACS v1.4.2 (Zhang et al., 2008) and underlying sequence comparison done with XXmotif (Hartmann et al., 2013). Generation and normalization of read depth profiles was performed in Bioconductor (Gentleman et al., 2004).

For further details, please see Extended Experimental Procedures.

Extended Experimental Procedures **Cell Lines**

HEK293, HEK293T-Rex and derived cell lines were grown in a humidified incubator with 5% CO_2_ in DMEM medium, supplemented with 10% FCS, 1% glutamine, and 1% penicillin/streptomycin (final concentrations). In order to establish a doxycycline-inducible RECQL5-expressing cell line, HEK293T-Rex (Invitrogen) were transfected with pcDNA4/TO-RECQL5-FL plasmid, expressing full-length RECQL5 under a tetracycline/doxycycline responsive promoter, and Zeocin-selected. Doxycycline (Sigma) was added at the indicated concentrations.

For the production of viral particles containing the HIV-derived vectors, HEK293T cells were cotransfected according to the manufacturer instructions with the Trans Lentiviral Packaging Kit and control- as well as RECQL5-specific shRNAs (TRCN0000051414, named shRNA♯4; TRCN0000051417, named shRNA♯7; and TRCN0000051415, named shRNA♯5, and TRC Lentiviral pLKO.1 Empty Vector Control, here named shCTR, all from Thermo Scientific), in 15 cm dishes using calcium phosphate. 48 hr after transfection, viral supernatant was collected, filtered and added to HEK293 cells. The knockdown efficiency was assessed by immunoblotting, using anti-RECQL5, and anti-tubulin or anti-Actin (Abcam) used for loading controls.

For generating a stable cell line in which endogenous RECQL5 was depleted so that virtually all RECQL5 was from the doxycycline-regulated gene, the pcDNA4/TO-RECQL5 FL plasmid was mutagenized to make it resistant to shRNA♯4 (shRNA♯4-Res primer sequence: 5′-ttcctggtgcagactttactattcaagaaacgaccgggacc-3′), This form of RECQL5 was stably expressed in HEK293T-Rex cells that were subsequently infected with Lentiviral Particles carrying the shRNA♯4 construct to deplete endogenous RECQL5. Single colonies were selected with puromycin in the presence of 0.2 ng/ml of doxycycline to allow physiologic expression level of the shRNA resistant version of RECQL5. Single colony-derived populations were grown in doxycycline and assessed by immunoblot analysis 4–5 days after doxycycline removal to test for remaining RECQL5, and for the expression of physiological levels of RECQL5 in the presence of doxycycline. Two independent clones were selected, and used in all experiments.

**ChIP-Seq**

For ChIP experiments, cells were harvested by trypsin-treatment and fixed in suspension with formaldehyde, 1% final concentration, for 15 min (min) at room temperature, with rotation. The crosslinking reaction was quenched with glycine (125 mM final concentration) for 5 min. Cells were washed twice with ice-cold 1× PBS and lyzed in 1 ml of ChIP cell lysis buffer (5 mM HEPES pH 8.0, 85 mM KCl, 0.5% NP-40, and protease inhibitors) and incubated 5 min on ice. Nuclei were pelleted by centrifugation at 3,900 g for 5 min at 4°C. Finally, nuclei were lyzed in ChIP nuclear lysis buffer (50 mM Tris-HCl pH 8.1, 10 mM EDTA (pH 8.0), 1% SDS, and protease inhibitors) and incubated 5 min on ice. Nuclear lysate was sheared by using an ice-water bath-embedded Bioruptor sonication system at high power, 30 s on, 30 s off mode for 5–10 min. The size of the sheared DNA was checked by 2% agarose gel electrophoresis to be between 300–400 base pairs (bp). Sonicated chromatin was cleared by centrifugation at 20,000 g for 15 min at 4°C. Before the immune-precipitation, chromatin was diluted 1:5 with ChIP dilution buffer (0.01% SDS, 1.1% Triton X-100, 1.2 mM EDTA (pH 8.0), 16.7 mM Tris-HCl pH 8.1, 167 mM NaCl and protease inhibitors). 1 μg of RNAPII antibody (4H8; recognizes all forms of RNAPII), or 1 μg of mouse IgG (Sigma), was bound to 15 μl of Protein A Dynabeads (Invitrogen) in 200 μl 5% BSA in PBS for 1 hr (h), before being washed twice with 500 μl of the same buffer. The sonicated chromatin was incubated with the antibody-conjugated beads overnight (o/n) at 4°C with rotation. Beads were washed twice with 1 ml of each of the following buffers: ChIP low salt buffer (0.1% SDS, 1% Triton X-100, 2 mM EDTA, 20 mM Tris-HCl pH 8.1, 150 mM NaCl); ChIP high salt buffer (0.1% SDS, 1% Triton X-100, 2 mM EDTA, 20 mM Tris-HCl pH 8.1, 500 mM NaCl); and ChIP LiCl buffer (10 mM Tris-HCl pH 8.0, 250 mM LiCl, 1% NP-40, 1% deoxycholic acid, and 1 mM EDTA). Beads were washed once with 1 ml of TE buffer (pH 8) and centrifuged for 1 min at 14,000 g before removing the buffer. Beads were finally suspended in 40 μl Elution Buffer (50 mM Tris-HCl pH 8.0, 10 mM EDTA, 1% SDS) and incubated at 65°C for 15 min. The eluted ChIP material was incubated at 65°C o/n to revert the crosslinking with an additional 90 μl of TE 1×/SDS1% and 1 μl 10 mg/ml RNase A. In parallel, the whole-cell extract was also RNase treated and reverse crosslinked o/n at 65°C. Proteinase K (100 μg) and Glycogen (20 μg) were added to the eluted ChIP material and incubated for 2h at 37°C, and DNA was twice extracted with phenol-chloroform-alcohol isoamylic acid, and precipitated with ethanol/NaCl. Precipitated DNA was submitted for further manipulation by standard ChIP-seq library preparation techniques (Illumina) and Advanced Sequencing on an Illumina GA IIx DNA sequencer. 36 bp single-end reads were aligned to the hg19 genome assembly using Bowtie 0.12.7 (Langmead et al., 2009) with the “–best” option. BAM files were merged, and reads that mapped to regions DAC blacklisted for mappability by the Encode project were removed. BAM files were sorted and indexed using SAMtools (Li et al., 2009). Further analysis was conducted using Bioconductor (Gentleman et al., 2004). Reads were extended to 200 bp.

**TSS Profiles**

Read depth coverage over the region 500 bp upstream to 2,000 bp downstream of the transcription start site (TSS) of all Ensembl protein coding transcripts was calculated, then normalized such that each sample had a total depth equivalent of 20 million reads (a similar number of reads was obtained for all compared samples; in the 40 million range for the KD experiments, and 25–30 million range for RECQL5 overexpression and its control). The mean normalized read-depth over each bp position was used to construct a plot of the average TSS profile.

**Traveling Ratios**

Traveling ratios were calculated as in (Rahl et al., 2010). Briefly, each transcript was divided into i) a promoter-proximal bin −30 bp to +300 bp around its TSS and ii) a gene body bin to the TTS. The traveling ratio is the ratio of RNA Pol II density in the promoter-proximal bin to that in the gene body. For the purposes of visualization, transcripts for which a travel ratio was calculated to be zero (i.e., no reads in promoter) or infinite (i.e., no reads in gene body) were removed from the analysis. The points plotted in Figures 1D, S1C, and S1F are the log base 2 travel-ratios, though the numbers on the axis are given in a linear scale, similar to previous studies (Rahl et al., 2010).

**Ratio of Traveling Ratios**

For the ratio of traveling ratios (Figure 1D), we created a subset of all protein coding Ensembl transcripts with an associated EntrezID based on a set of filters previously described by others (Brannan et al., 2012). More specifically, transcripts arising from neighboring genes that lay within 2 kb of one another and also transcripts that spanned <2 kb or >300 kb of genomic space were removed. Finally, we subset what remained by insisting that any given transcript must have an RNAPII peak called over its TSS in at least 2/3 of the RECQL5 depletion samples and at least in one of the Doxycycline samples, this left us with a total of 5,140 unique Ensembl transcripts. The ratio of traveling ratios was defined as the traveling ratio over a specific gene in RECQL5 depleted (or overexpressing) cells, divided by the traveling ratio of its respective control on the same gene. The number of instances of the ratio of traveling ratios (RoTR) being greater or less than 1 in the RECQL5 overexpressed and knockdown samples was counted. A hypergeometric test was used to calculate the significance of the large overlap between the genes showing a >1 RoTR in the RECQL5 depleted sample and <1 RoTR in the RECQL5 overexpressed sample.

**Correlation with Expression Data**

Total RNA was extracted from RECQL5-expressing control cells (0.2 ng/ml doxycycline) and from the same cells depleted of RECQL5 96 hr after doxycycline washout, using the RNeasy Mini Kit (QIAGEN). 20 μg of total RNA was reverse transcribed using oligo-dT primers with the cDNA Synthesis System, according to the manufacturer’s recommendations (Roche). 1 μg of the synthesized cDNA was hybridized on a 12X135K Human Expression Array HG18 (Nimblegen-Roche), with labeling, hybridization, and washing performed according to manufacturer’s recommendations (Nimblegen-Roche). Arrays were scanned with a Nimblegen MS200 micro-array scanning system and images were processed with Nimblescan according to manufacturer’s recommendations (Nimblegen-Roche). Acquired image data were processed with the DNAStar ArrayStar software (DNASTAR) and transcripts with a >1.5-fold change between the WT and the KD average expression values and p value < 0.05 were used together with their t-statistics. Gene expression data from Nimblegen arrays were merged with the ratio of traveling ratios calculated for the shRNA7 RECQL5 depletion sample. Where multiple transcripts existed on the array, the one with the largest absolute t-statistic was selected. The significance of the enrichment between gene expression t-statistic and the ratio of traveling ratio was assessed using a mean-rank gene set test. The p value was >0.7, suggesting that there was no significant association between the ratio of traveling ratios and differential gene expression. Spearman’s correlation coefficient between the t-statistic and the traveling ratios was −0.05, also suggesting negligible correlation.

**DRB/GRO-Seq**

Elongation rate experiments (Figures 2B and 2C) were carried out as described (Singh and Padgett, 2009). Briefly, cells were treated for 3.5 hr with 100 μM DRB (Sigma) to inhibit transcription, then washed twice in PBS, and incubated with fresh complete medium for transcription restart. Every 5 min a cell dish was lysed directly in RLT buffer + β-Mercaptoethanol and collected on ice with a scraper. RNA was extracted with RNeasy MiniKit (QIAGEN), according to the manufacturer’s recommendations. 2 μg of Total RNA were Reverse Transcribed with random hexamers with the TaqMan Reverse Transcription Kit (Applied Biotechnology-Invitrogen). Pre-mRNA levels were assessed by quantitative RT-PCR using iQ-SYBR Green Supermix and CFX96 Real-Time System (Biorad); the ratio relative to the control sample was plotted. Primers used in the quantitative RT-PCR are the same as previously shown (Singh and Padgett, 2009). Results shown are average values of a minimum of three independent replicates, ± standard error.

For DRB/GRO-seq, a 15 cm dish at 80%–90% of confluence was processed at each time point for nascent RNA mapping. Cells were initially treated with DRB for 3.5 hr as described above, and samples from time points 10, 25, and 40 min after release into fresh medium were processed. Transcription-competent nuclei were prepared using the Nuclei Isolation Kit according to the manufacturer’s recommendations (Sigma). Nuclear Run-On reactions were carried out with Br-UTP as described (Core et al., 2008), and Br-UTP run-on labeled RNA was isolated using Br-UTP-specific antibody (IIB5, Santa Cruz). The purified RNA was used for the preparation of strand-specific RNA libraries using standard Illumina protocols, and sequenced on an Illumina GA IIX sequence analyzer. 36 bp single-end reads were aligned to the hg19 genome assembly using BWA v0.5.9 (Li and Durbin, 2009) with a seed length of 36 (-l 36), allowing up to 3 alignments per read (-n 3). BAM files were merged, and reads mapping to Ensembl rRNAs, or to regions DAC blacklisted for mappability by the Encode project, were removed. BAM files were sorted and indexed using SAMtools (Li et al., 2009).

Further analysis was conducted using Bioconductor (Gentleman et al., 2004). Reads were extended to 250 bp, and each sample was normalized to a read depth of 20 million. A subset of the protein coding human Ensembl transcriptome was created by filtering for transcripts ≥30 kb. The largest transcript per gene was selected, resulting in a list of 8,529 transcribed genes. Base pair level coverage of the region 2 kb upstream, to 120 kb downstream, of each transcript’s TSS was calculated for each sample. Average transcript profiles were generated by taking a trimmed mean (0.01) of read depth over each base pair. The normalized read depth across the CTNNBL1 gene was smoothed using the smooth.spline function from Bioconductor’s stats package (spar = 0.8).

**Wave Fronts**

Regions in which the normalized read depth was ≥3 bp were identified for each gene from the TSS to 120 kb downstream. These regions were assumed to be evidence of elongation. When read-depth first dropped below 3 for 5,000 consecutive base pairs, elongation was assumed to have halted, and a wave front was called at the transition point. The 5 kb distance filter was necessary to filter out background noise and downstream transcripts. Because of low-level contamination with stable mRNAs in the samples, as previously described by others (Schwartz et al., 2011), the short exonic regions were also excluded in the wave front calling analyses. Examples of wave front calling are shown in Figure S2A.

**Elongation Rates Based on Wave Fronts**

The transcripts from above were further filtered such that (1) a wave front must have been successfully called in all 8 (4 time points, KD + WT) samples from a given cell type, (2) the mean normalized read depth in the promoter (−2 kb/+120 kb of the TSS) was at least 10 in both WT and KD at time point 0, (3) the mean coverage in the promoter region was larger than for the whole 122 kb region surveyed, iv) the wave front advanced with time in both the WT and KD cells (i.e., t40 > t25 > t10 > t0). Because of these strict requirements, this left only distinct 237 genes/transcripts (shown in Figure 4C).

Elongation rates were then calculated by dividing the difference in wave front positions at two time points by the number of minutes that separated them. The significance of the difference between the increased elongation rates in the KD sample relative to WT was assessed using a one-side, paired Wilcoxon test. Transcripts clearly showing multiple TSSs in the body of the transcripts were excluded by the analyses because it led to a miscalling of the wave front. Only transcripts ≥100 kb (n = 103) were considered for statistical testing, except for the rate calculated between the early 25 and 10 min time points, where a ≥50 kb filter (n = 177) was applied. This was required to ensure that all genes included in the calculations were long enough for transcript elongation not to have reached the TTS at the time of measurement. The box-plot representation of the calculated values (Figure 4D) show median values ± 25% quartiles in the box and minimum/maximum distribution of the values in the whiskers.

**RNAPII Peak Calling**

Peaks were called against an IgG control using MACS v1.4.2 (Zhang et al., 2008) with the following settings:–gsize (hs),–tsize (36),–mfold (8,30),–pvalue (0.00001). Around 12k peaks were called in wild-type cells, 30k in shRNA5-treated cells, and 40k in shRNA7-treated cells.

Peaks in annotated human transcripts. There are 81,623 Ensembl protein-coding transcripts that map to a single location on Chr1-22,X,Y,M. In shRNA7-treated cells, 37,042 of these contained a promoter-proxmial RNAPII peak *and* at least one peak in the transcribed region, while 2,267 only had a peak in the transcribed region (i.e., TSS+500 bp to TTS). This corresponds to 48.15% of annotated Ensembl transcripts (39,309/ 81,623). Because of the occurrence of genes-within-genes, more than one annotated transcript can contain the same RNAPII peak, helping explain why so many overlap.

**Peak Enrichment Ratio**

The number of RNAPII peaks overlapping Ensembl protein coding transcripts’ TSS (±500 b) and gene bodies (+500 b to TTS) were counted. The ratio of peaks called in the gene bodies to peaks called in the TSS were calculated to look for enrichment of the polymerase in different portions of the gene.

**RNAPII Read Density Surrounding MACS Peaks**

Sample coverage was normalized to a depth equivalent to 20 million reads. For each MACS peak, 300 genomic intervals covering the region 2 kb upstream of each peak, the peak itself and the region 2 kb downstream were created. The upstream and downstream regions were both 2 kb in size and split into 100 equally sized intervals. The peak regions themselves were of an inconsistent width, but were likewise binned into 100 equally sized intervals. For each peak, the mean normalized read depth across each of the 300 intervals was calculated. Finally, a mean across each interval for all peaks was taken and plotted.

**Characterization of Transcription Stress Sequence Features/Signatures**

Overrepresented sequence consensus elements were searched for with XXmotif (Hartmann et al., 2013) (settings:–zoops–type ALL–localization–merge-motif-threshold LOW) in ±50 and ±400 bps surrounding RNAPII peaks occurring in the transcribed regions, excluding the first 500 bp of genes (to avoid motifs associated with the transcription initiation). For peaks in transcripts on the negative strand we searched in the reverse-complement the positive-strand reference genome sequence.

To analyze the enrichment of oligonucleotides around RNAPII peaks over and above what is expected from the bias in mononucleotide composition around these peaks, we computed position specific log-odds ratios between the oligonucleotide frequencies and their expected frequencies based on the mononucleotide frequency profiles (Figure S3A). The logg-odds ratio profiles do not show changes near the RNAPII peaks and are similar to other transcribed regions. Therefore, oligonucleotide composition is already accounted for by the mononucleotide composition profiles around RNAPII peaks.

For Figure 6 we counted and summed the frequencies of A and T nucleotides in a ±1 kb region around the RNAPII peaks and plotted them as histograms grouped by different AT frequencies. For the exon and intron subfigures we sampled a similar number of random positions in exons and introns, respectively, excluding any positions closer than 500 bps to a transcription start site, to match the selection criteria of the RNAPII peaks.

**Comparative Genomic Hybridization**

Two, independently derived doxycycline-responsive RECQL5 knockdown cell lines were grown in parallel for 10 days with or without 0.2 ng/ml of doxycycline. Genomic DNA was then isolated using the QIAamp DNA kit (QIAGEN), according to the manufacturer’s recommendation. The purity of the DNA was verified by a NanoDrop spectrophotometer (Thermo Scientific) to be A260/280 > 1.8, and A260/230 > 1.9. The integrity of DNA was also verified by agarose gel electrophoresis, with no RNA contamination observed. Nimblegen’s (Roche) 3 × 720K human arrays were used for the experiments. The labeling of the samples, hybridization, and washing was performed according to the manufacturer’s recommendations (Nimblegen-Roche). The array was scanned by the Nimblegen MS200 micro-array scanning system (Nimblegen-Roche). Primary data extraction, analysis, and visualization were performed using the DEVA software with the default settings for “CGH Workflow with segmentation” (http://www.nimblegen.com/products/software/deva/index.html, Roche). Briefly, this included LOESS spatial correction, Qspline normalization (Workman et al., 2002), and segMNT v1.1 analysis, as employed by DEVA. Only losses and gains encompassing 4 or more probes and a mean log value of ≥0.2 were considered.

In order to calculate the correlation between the identified CGH breakage regions and transcription, gene start- and end-points were identified using Ensembl GRCh37, and the number of CGH regions that overlapped by at least one base with a gene was calculated. This provided an observed proportion of regions containing a gene. We then bootstrapped a null distribution, by randomizing the CGH regions (within their original chromosome and maintaining region size), by selecting a random CGH probe as the new starting point. We repeated the calculation of proportions of regions containing a gene, and bootstrapped n = 10,000 to obtain empirical “expected values” and p values that a random set of regions would, more often than observed, contain genes. We repeated this analysis for different subsets of the CGH data (lost/amplified/all), and different subsets of the Ensembl data.

For analyzing in which part of a gene/transcript the breakpoint occurred, we extracted the start- and end-points of the CGH regions as a set of breakpoints, and calculated the number of breakpoints that were contained within a gene. This observed number was then tested against a null model of random breakpoints across the genome, by a binomial test where the probability of a single breakpoint being contained in a gene is the proportion of the genome covered by genes, and the number of trials in the test is the number of breakpoints in total.

For the correlation between the CGH breakage regions and chromosome fragile sites (CFSs), we used the previously described randomized approach comparing the CGH regions with the aphidicolin-induced CFS region list as presented in Figure S1 of (Fungtammasan et al., 2012).

**Overlap of Genome Instability and RNAPII Peaks**

Proximity between points of genomic instability and loci of RNAPII peaks was assessed by, for each breakpoint in each of the three experimental conditions, measuring its absolute distance to its nearest RNAPII peak (for Figure 7A restricting the peaks to being in the same gene as the breakpoint; 7B having no such restriction). To get null distributions of these distances per experiment, we computer-randomized the breakpoints across the locations the array had probes for, preserving the number of inter- and intragenic breakpoints in each experiment. Randomization was performed 100 times. The distances within each randomization were ranked, and the mean value for each centile across randomized breakpoints gives the “random” number plotted – allowing an assessment of whether the observed distances are closer than expected by chance. To assess significance of the observed abundance of genes with both peaks and breakpoints, a Poisson test of the number of instances of peaks in genes with breakpoints was carried out against the background rate of peaks in genes without breakpoints.

## Author Contributions

M.S. performed the experiments, with contributions from T.K. (CGH) and H.W. R.M., G.P.K., and A.S. performed most bioinformatics and statistical analysis, but ChIP-seq peak sequence analysis was done by M.H. and J.S. J.Q.S. wrote the manuscript, with help from all coauthors.

## Accession Numbers

The GEO accession number for the genome-wide data-sets reported in this paper is GSE49134.

## Supplemental Information

Table S1. List of Transcripts that were Down- or Upregulated in Response to RECQL5 Knockdown, Related to Figure 1

Table S2. Characteristics of the 161 Examples of Genomic Loss and 88 Examples of Gain Observed upon RECQL5 Knockdown, Related to Figure 5

Table S3. Genomic Rearrangements Observed in Wild-Type Cells, CGH-WT versus WT; Total Observed in Two Independent Experiments, Related to Figure 5

Table S4. Genes and Transcripts in the 161 Examples of Genomic Loss and 88 Examples of Gain Observed upon RECQL5 Knockdown, Related to Figure 5

Document S1. Article plus Supplemental Information
